# No evidence of parental mental health influencing children’s academic achievement: a within-family study

**DOI:** 10.1007/s00787-026-03100-9

**Published:** 2026-06-24

**Authors:** Perline A. Demange, Michel G. Nivard, Fartein A. Torvik, Espen M. Eilertsen, Rosa Cheesman, Magnus Nordmo, Torkild Lyngstad, Eivind Ystrom, Elsje van Bergen

**Affiliations:** 1 PROMENTA Research Center, Department of Psychology, University of Oslo, Oslo, Norway; 2 Department of Biological Psychology, Vrije Universiteit Amsterdam, Amsterdam, The Netherlands; 3 Research Institute LEARN!, Vrije Universiteit Amsterdam, Amsterdam, The Netherlands; 4 Amsterdam Public Health Research Institute, Amsterdam, The Netherlands; 5 Population Health Sciences, Bristol Medical School, Bristol, UK; 6 MRC Integrative Epidemiology Unit, University of Bristol, Bristol, UK; 7 Centre for Fertility and Health, Norwegian Institute of Public Health, Oslo, Norway; 8 PsychGen Center, Norwegian Institute of Public Health, Oslo, Norway; 9 Department of Educational Science, University of South-Eastern Norway, Notodden, Norway; 10 Department of Sociology and Human Geography, University of Oslo, Oslo, Norway; 11 Centre for Research on Equality in Education, University of Oslo, Oslo, Norway; 12 Department of Medical Epidemiology and Biostatistics, Karolinska Institutet, Stockholm, Sweden

**Keywords:** Parental mental health, Academic achievement, Educational attainment, Children-of-siblings design, Within-family design, Familial confounding, Genetic confounding, Quasi-experimental design, Intergenerational transmission, Parental psychopathology

## Abstract

Children of parents with psychopathology generally perform less well in school than their peers. Parental symptoms might not, however, account for their lower achievement, as other familial factors might contribute. To examine the role of parental symptoms, we analyse data from 9,000 families in the Norwegian Mother, Father, and Child Study (MoBa). Parents reported their symptoms of anxiety, depression, ADHD, eating disorders, and alcohol use disorder during pregnancy and during their child’s childhood. Children in 5th grade (aged 10) completed nationally-standardised tests of mathematics, reading comprehension, and English (as an additional language). Comparing children who are cousins (children-of-siblings design), we controlled for unmeasured factors shared among family members that confound the relationship between parental mental health and children’s academic achievement. We found small associations between greater parental symptoms and lower scores in reading and mathematics (− 0.060 ≤ β ≤ −0.013). However, these associations were no longer significant when comparing cousins whose parents differ in mental health. Results suggest that parental symptoms do not exert a direct, clinically meaningful effect on children’s academic achievement. Although non-significant, associations with parental symptoms measured close to the child’s academic test are less attenuated than those measured during pregnancy. Notably, comparison between the overall MoBa sample and the children-of-siblings samples suggests ascertainment differences that hinder strong generalisations. Our findings underscore the importance of quasi-experimental family designs in identifying modifiable targets in intergenerational health and education research. Supporting parental mental health is crucial but its direct effect on children’s achievement may be smaller than often thought.

**Supplementary Information:**

The online version contains supplementary material available at https://doi.org/10.1007/s00787-026-03100-9.

## Introduction

A significant proportion of children live with parents suffering from mental disorders: recent assessments in western countries suggest 10 to 25% of children have an affected parent [1, 5, 43, 50]. Children of parents with mental disorders are a particularly vulnerable population. Parental psychopathology is associated with childhood adversity [50] and poorer child health, behavioural and academic outcomes [28, 31, 52].

Overall, previous studies suggest that poor parental mental health is associated with poor educational outcomes in children. However, evidence for a negative association is highly inconsistent across studies and might depend on the specific parental mental health problem, its severity, time at measurement, children’s outcomes, parent sex, study population, etc. Internalizing disorders (such as depression and anxiety) are among the most frequently studied parental risk factors in this regard, and studies report varying degrees of null [4, 22, 47, 48] to negative [4, 6, 9, 40, 45, 48, 60] associations with children’s academic achievement. Beyond internalizing disorders, other parental mental health conditions also show mixed effects. Having a parent with schizophrenia appears to negatively affect children’s academic performance, while having a parent with bipolar disorder does not [40, 53]. The effect of parental eating disorders has yielded divergent results [42]. The association with alcohol and substance use is also unclear and might depend on timing of problematic usage and quantity [39, 63].

All these studies report observational parent-child associations, adjusted for a limited number of measured covariates. While identifying specific negative associations between parental mental health problems and academic achievement allows us to identify at-risk groups, identifying causal effects is necessary to understand the mechanisms underlying parent-child transmission and evaluate potential interventions. Observational parent-child associations are likely to be confounded by shared familial factors, such as socioeconomic position, neighborhood environment, shared adverse experiences, cultural norms, or genetic influences [13, 27, 60]. As a result, observed associations may reflect these confounding familial influences rather than a direct environmental effect of parental symptoms. Within-family designs reduce confounding from genetic and environmental factors shared within family members, without requiring these factors to be directly measured [19]. To our knowledge, only two studies have used such designs to study the effect of parental mental health on children’s education, looking at maternal smoking [38] and parental schizophrenia [32]. Both find no evidence for a causal effect on children’s school performance.

**Fig. 1:**
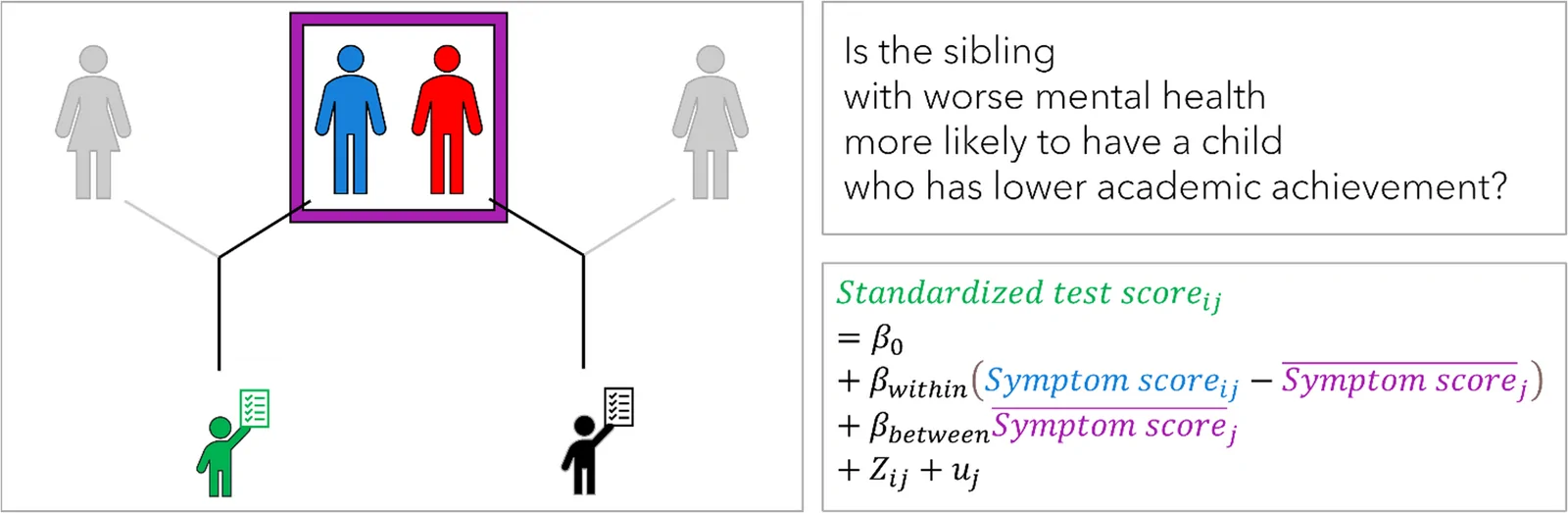
Visual summary of the children-of-siblings design. The research question we aim to answer with this design is “Is the sibling with worse mental health more likely to have a child who has lower academic achievement?”. We present the regression model used and a drawing illustrating the family members of interest. We colour-coded for clarity: the outcome of interest is the standardised test score of the child (green), the predictors are the average symptom scores of the parent and their siblings (purple), and the deviation of the parental symptom score (blue) from the sibling average. $\:{Standardized\:test\:score}_{ij}$  is the standardised test score (academic achievement) of the child i. $\:{Symptom\:score}_{ij}$  is the symptom score of the parent of the child i. $\:{Z}_{ij}$  are covariates: sex and year of birth of the child, years of birth of the mother and of the father, sex of the parent. $\:{u}_{j}$  is the random effect for the family j (family including the parental siblings and their children). $\overline{Symptom\:score_j}$  is the average symptom score of the parent and their siblings (average score for the family j)

Within-family designs have the key advantage of not requiring the measurement of confounding factors: by comparing family members, the factors shared between these family members are held constant, providing an adjusted estimate of the association of interest. Which factors are controlled depends on which family members are compared. For our research question on intergenerational effects, we use a children-of-siblings design (Fig. 1), also sometimes referred to as a cousin comparison or cousin fixed-effect design [18, 21, 57]. In this design, we compare parents who are siblings, and their children who are cousins. We test whether a parent with more symptoms is more likely to have a child with lower academic achievement than their sibling. Comparing parents who are siblings adjusts for factors they share, including roughly 50% of their segregating genes and aspects of their childhood family environment. This therefore provides an estimate controlled for various unmeasured familial sources of confounding that would persist in conventional observational associations. Factors not shared between siblings (e.g. the remaining 50% of their genes, their adult environment) may still confound the association between parental symptoms and child achievement. Thus, the design does not by itself establish causality, but it strengthens inference about whether associations reflect a direct effect of parental symptoms rather than shared familial influences. Furthermore, if the within-sibling estimate is smaller than the observational estimate, this suggests that factors shared within the extended family partly explain the observed association. A causal diagram illustrating the design can be found Supplementary Fig. 1, as well as a discussion of the role of genetic liability in Supplementary Note.

In this study, we investigate the role of parental symptoms leveraging data from up to 9,000 families of the Norwegian Mother, Father, and Child Study (MoBa). Parents filled out surveys on their symptoms of anxiety, depression, eating disorders, ADHD, and alcohol use disorder. Children in 5th grade (aged 10) participated in nationally-standardised tests of mathematics, reading comprehension, and English (as an additional language). Comparing children who are cousins allows us to control for unmeasured familial factors (e.g., part of the genetic influences and socioeconomic background) that confound the relationship between parental mental health symptoms and children’s academic achievement. 

## Methods

The study was preregistered; the registration is available on the Open Science Framework website: https://osf.io/sqz3n/. Deviations from the pre-registration are stated in the Supplementary Note.

All code associated with the analyses is available on GitHub at https://github.com/PerlineDemange/ParentMH_childEA_Moba.

### Sample

We use data from the Norwegian Mother Father and Child Study (MoBa). The Norwegian Mother, Father and Child Cohort Study (MoBa) is a population-based pregnancy cohort study conducted by the Norwegian Institute of Public Health. Participants were recruited from all over Norway from 1999 to 2008. The women consented to participation in 41% of the pregnancies. The cohort includes approximately 114,500 children, 95,200 mothers and 75,200 fathers. The current study is based on quality-assured data files released for the project dated 13.05.2023. The establishment of MoBa and initial data collection was based on a license from the Norwegian Data Protection Agency and approval from the Regional Committees for Medical and Health Research Ethics. The MoBa cohort is currently regulated by the Norwegian Health Registry Act. The current study is part of the project “2017/2205 Sosial ulikhet i Norge og barns psykiske utvikling (SUBPU)” [Social inequality in Norway and children’s psychological development], which was approved by the Regional Committees for Medical and Health Research Ethics (REK).

We estimate associations in the full sample of the Norwegian Mother Father and Child Study (MoBa) using available measures, and within-family associations in a subset thereof (referred to as the children-of-siblings sample). We used linkage to the Norwegian central population register for basic demographic data and kinship links. Data availability for research purposes is subject to strict Norwegian privacy regulations, see https://www.fhi.no/en/studies/moba/for-forskere-artikler/research-and-data-access/ and https://www.ssb.no/en/data-til-forskning/utlan-av-data-til-forskere.

Within the Norwegian central population register, we identified all children sharing the same legal mother and father. As the children-of-siblings design relies on the assumption that siblings and cousins are genetically related, we excluded children with same-gender parents, because in these families, registry-based legal parentage does not consistently identify biological parentage. Gender was here defined as the legally registered gender in the Norwegian population register in 2023. We then identified children whose mothers are sisters (i.e. these children are maternal cousins, they share maternal grandparents), whose fathers are brothers, and whose parents are siblings of the opposite gender. Linking this information on cousins and parental siblings, we selected in MoBa parents for whom at least one full sibling (including twins) also participated in MoBa. These parental siblings and their children were included in the analysis if both siblings reported mental health and at least one of their children has data available for the national academic test in 5th grade. We only included one child per parent. Some children were part of multiple extended families: once via their mother, once via their father. We excluded one of these familial links at random to avoid duplicated children. Our main analyses use the full sample of cousins in the child generation based on maternal, paternal and cross-sex parent siblings to maximize the sample size. Sex here, and in all analyses, is defined as sex (self- and mother-) reported in MoBa questionnaire at birth. Flowchart of the sample construction is presented in Supplementary Fig. 2 and final sample sizes are presented in Table 1.

## Measures

### Academic Achievement

The child academic outcome variables are individual scores on national standardised tests in 5th grade. Pupils take national standardised tests in 5th, 8th and 9th grade in mathematics, reading comprehension (in their native language: Norwegian) and English as an additional language. We focus on 5th grade (~ 10 years-old) as it is before the typical age of onset of many psychopathologies and is the academic achievement with the largest available sample size linked to MoBa. The test scores are obtained from linkage with the National Educational database (NuDB) via Statistics Norway. We standardised the scores within each test and year combination, in the overall NuBD population, so that our outcome measures how the child places themselves in their own cohort on the specific test [30]. For pupils who took the tests two years in a row, we only considered the results obtained the first year.

### Parental mental health

#### Timing

Parents in MoBa completed extensive questionnaires about their health, lifestyle, and work. For our analyses, we included all available well-validated self-report symptom scales assessing current mental health, covering anxiety, depression, ADHD, alcohol use disorder, and eating disorders. Surveys for mothers and fathers were not always collected at the same time, so we describe timing for each symptom measure. As a rule of thumb, we selected parental measures assessed at 15 weeks of pregnancy (first MoBa survey and only survey sent simultaneously to both parents) and the closest in time to the child passing the national test (age 10). While mothers were surveyed regularly following child’s age, father were only surveyed during pregnancy and in 2015. The 2015 questionnaire was sent regardless of the children’s age. For symptoms measured with the 2015 questionnaire, we only included the subset of fathers whose children were younger than 10 in 2015, to ensure that the symptom was measured before the child’s national test.

For each measure of mental health in parents, we run a series of models to test the association with childhood educational test scores. Each model aims to further control for measured and unmeasured confounding, through sibling comparison in the parental generation. We standardised the mental health scores with a variance of 1 and mean of zero before each model. For each model, we adjust our p-values for multiple testing, using a false discovery rate correction. All statistical tests are two-sided. Analyses were done with R.4.2.3.

We first ran a mixed-effects regression to estimate the minimally adjusted association between the parental mental health traits and the child’s academic achievement. We ran this model (1) in the full MoBa parent-child couples with available measures. We also ran (1) in the children-of-siblings subset, to serve as a comparison baseline to the children-of-siblings design, as subtle sample differences between the full sample and the children-of-siblings subset might affect estimates.

1 $$\begin{array}{c}{Standardized\:test\:score}_{ij}=\:\\{\beta\:}_0+\:{\beta\:}_1{Symptom\:score}_{ij}+\:Z_{ij}+u_j\end{array}\:\:$$

$\:{Standardized\:test\:score}_{ij}$ is the standardised test score (academic achievement) of the child i. $\:{Symptom\:score}_{ij}$ is the symptom score of the parent of the child i. $\:{Z}_{ij}$ are covariates: sex and year of birth of the child, years of birth of the mother and of the father, sex of the parent. $\:{u}_{j}$ is the random effect for the family j (family including the parental siblings and their children).

In the children-of-siblings sample, we estimated the intraclass correlation of symptom scores in parental siblings. This reflects the proportion of total variance in mental health that is attributable to differences *between* sibling pairs, so an indication of how similar the siblings are to each other.

To estimate the association between the parental symptom score and the child’s school performance adjusted for unmeasured shared familial factors, we then ran the children-of-siblings design: a regression including within and between-family effects of parental mental health on child’s academic achievement, effectively using the siblings of parents as de facto matched controls.

2 $$\begin{array}{c}{Standardized\:test\:score}_{ij}=\\{\beta\:}_0+\:{\beta\:}_{within}\left({Symptom\:score}_{ij}-\overline{Symptom\:score_j}\right)\\{+\:\beta\:}_{between}\overline{Symptom\:score_j}+\:Z_{ij}+u_j\end{array}\:\:$$

With $\overline{Symptom\;score_j}$ being the average symptom score of the parent and their siblings (average score for the family j).

The within-sibling effect represents the expected change in educational performance given one unit change in the difference between the parent’s mental health and the average mental health of the parents’ sibling pair. It represents the effect of parental mental health on the child’s test score while keeping factors common to the extended family constant.

In both models, we use random effects for the extended family (with a grandparental ID) to adjust standard errors for familial clustering, as we do not limit the number of parental siblings in our analyses (we do limit the analyses to one random child per sibling).

We also investigated the attenuation of the within-sibling effect when taking into account parental education, see Supplementary Note.

#### Sum scores for anxiety, depression, ADHD, and problematic alcohol use symptoms

Anxiety and depression symptoms were measured using subsets of the Hopkins Symptoms Checklist (SCL) [15], we use SCL-5 [62] at 15 weeks of pregnancy for both mothers and fathers, and SCL-8 at age 8 for mothers and 2015 for fathers.

ADHD symptoms were assessed with the Adult ADHD Self-Report Scale (ASRS Screener) [34, 35]. This was assessed during pregnancy for fathers and at 36 months for mothers.

Problematic alcohol use symptoms were measured with 7 items of the Alcohol Use Disorders Identification Test (AUDIT) [16, 58] which relate to problematic use (AUDIT-P). This was assessed at age 8 for mothers and in 2015 for fathers.

Sum scores for these scales were created following standard practice as implemented in MoBa and documentation of the questionnaires (Supplementary Note). We set a completion threshold of 0.75: 75% of the items had to be reported for the total score of the participant to be considered. Lower completion was considered a missing score. Extraction of the mental health results in MoBa was performed with the R package Phenotools (*Phenotools*, 2021/2023) version 0.2.9 [49].

#### Scores based on item response theory for eating disorder symptoms

Eating disorder symptoms according to the DSM-IV (*Diagnostic and Statistical Manual of Mental Disorders*,* 4th Ed*, 1994) [17] diagnostic criteria for anorexia, bulimia, and eating disorder not otherwise specified (i.e. purging disorder and binge eating disorder) were only assessed in mothers [10], during pregnancy and at age 8. With low prevalence of eating disorders and high level of comorbidity, we computed a continuous eating disorder risk score using item response theory on the DSM-IV eating disorder symptoms. In Stata 18, we computed a nominal response model for each time point, we report parameters estimates in Supplementary Tables 1 & 2 and test characteristics curve and test information functions in Supplementary Fig. 3.

## Results

### Descriptive statistics

Descriptive statistics for parental symptoms and child academic achievement are presented in Table 1 and Supplementary Table 3. Analytic sample sizes vary depending on missingness of the measures, from 36,313 to 76,672 in the overall MoBa (all parent-child pair) and from 2,358 to 17,868 for the children-of-siblings subsamples.

The average standardised test scores of MoBa children are above zero (0.10 to 0.35), indicating that children participating in MoBa have on average higher academic achievement than the Norwegian population (Supplementary Table 3). This is in particular for maths and reading scores, with average scores of 0.26 and 0.25 respectively, and even more pronounced in the children-of-siblings samples with average scores of 0.35 and 0.33.

Parents in the children-of-siblings samples had fewer symptoms and less variance in symptoms than the overall MoBa sample (Table 1). The intraclass correlations ranges from 0.07 to 0.15, reflecting modest sibling similarity: only 7–15% of the variation in symptoms is shared between siblings. This indicates that siblings frequently differ in their symptoms levels, which provides the variation required for sibling comparison models to estimate associations while accounting for shared family factors. For symptoms with measures at two time points, the intraclass correlation is higher in pregnancy than when the child is 8 years (0.13 vs. 0.07 for anxiety and depression symptoms and 0.15 vs. 0.08 for eating disorders symptoms), suggesting reduced familial clustering over time.

**Table 1:** Descriptive statistics of parental symptom scores at different time points in the children’s life. Average over the analytic samples (used in Fig. 2). Statistics per sample are in Supplementary Tables 3 & 4. Subsample “MoBa” refers to all parent-child pair with non-missingness in MoBa, and “CoS” refers to the children-of-siblings subsample of MoBa. Statistics regarding the mental health scores are before standardisation within the analytic sample. Intraclass correlation is between parental siblings

	Anxiety - Depression in pregnancy		Anxiety - Depression ~ age 8		ADHD ~ < 3yo		Alcohol Problematic Use ~ age 8		Eating disorder in pregnancy		Eating disorder ~ age 8	
Subsample	MoBa	CoS	MoBa	CoS	MoBa	CoS	MoBa	CoS	MoBa	CoS	MoBa	CoS
Scale	SCL-5		SCL-8		ASRS		AUDIT-P		IRT		IRT	
Sample N	77,111	17,868	42,322	4393	57,276	7205	42,244	4357	76,672	8237	36,313	2358
Mean	1.06	0.92	2.22	1.99	7.22	7.16	0.44	0.37	0.05	0.01	0.00	−0.03
SD	1.84	1.67	3.09	2.80	3.45	3.36	1.24	1.10	0.84	0.84	0.60	0.55
Skew	2.77	2.98	2.42	2.43	0.25	0.19	4.90	4.94	0.81	0.86	1.73	1.70
% girls	49	49	49	49	49	49	49	49	49	49	49	49
% mothers	59	57	69	67	64	65	69	68	100	100	100	100
Number of families	–	8700	–	2160	–	3540	–	2145	–	4042	–	1164
Intraclass correlation	–	0.13	–	0.07	–	0.11	–	0.08	–	0.15	–	0.08

### Observational associations

**Fig. 2:**
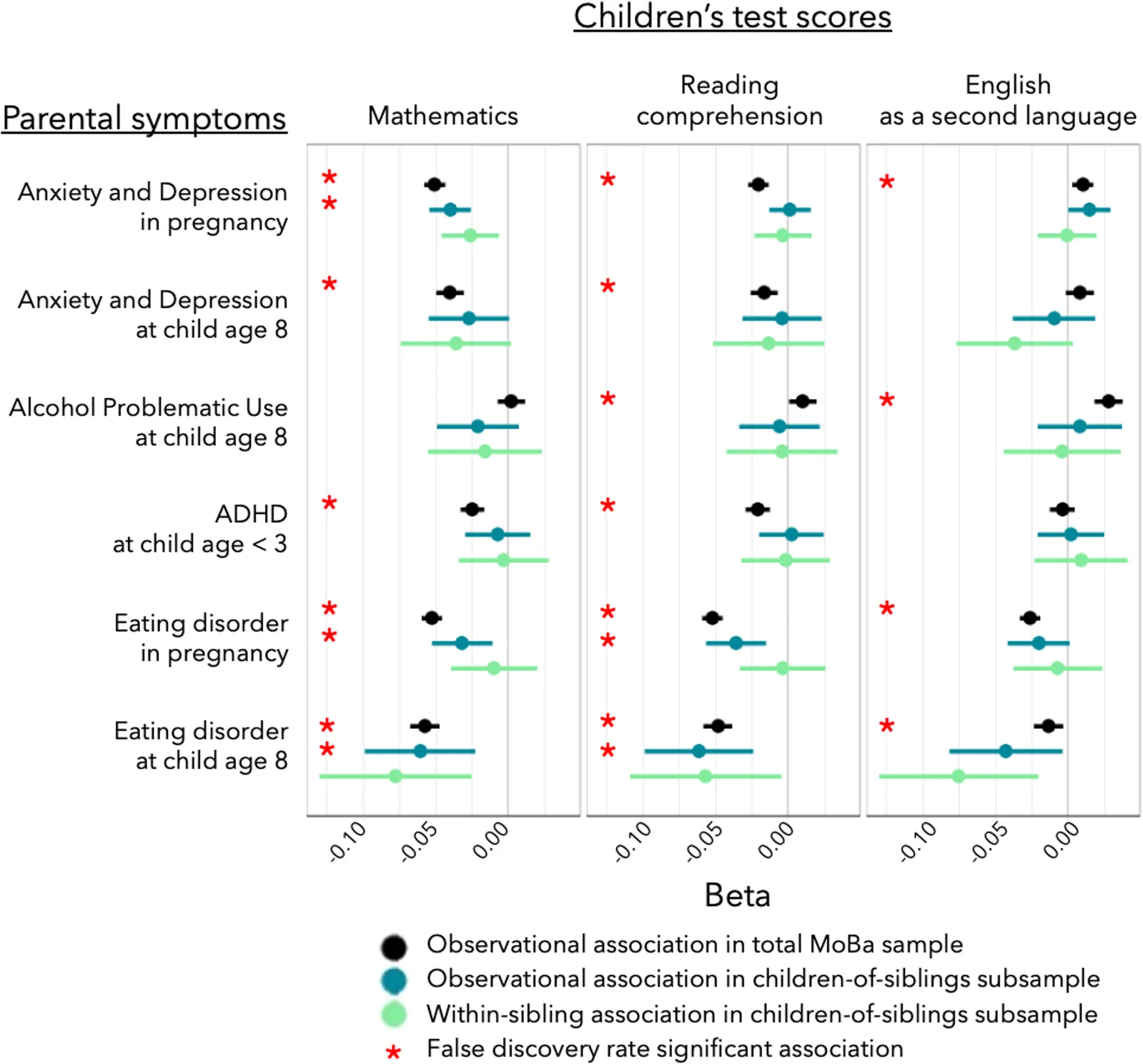
Association of parental symptom scores with children’s academic achievement. Coefficients as estimated with a linear regression in the total MoBa sample (black), in the children-of-siblings subsamples (blue), and with the children-of-siblings model (green). Red asterisks indicate estimates that reach statistical significance (p< 0.05) after false discovery discoveryrate correction for multiple testing. Bars are 95% CIs. Child ages at which parents reported their mental health are approximate (see Methods). Eating disorder symptoms were measured in mothers only

We first ran a linear regression to estimate observational associations between parental symptoms and children academic achievement. In the total MoBa sample, most parental mental health scores are negatively associated with their children’s academic achievement (Fig. 2 – black and Supplementary Tables 5 & 6). Parental anxiety and depression, ADHD and eating disorders symptoms are significantly negatively associated with scores in mathematics and reading tests, with standardised ßs from − 0.016 for anxiety and depression symptoms at age 8 with reading (SE = 0.00) to −0.057 for eating disorders at age 8 with maths (SE = 0.00). Postnatal alcohol problematic use stands out as it is not significantly associated with maths and is positively associated with reading scores (ß = 0.00, SE = 0.00 & ß = 0.01, SE = 0.00). Surprisingly, direction of associations with English test scores vary depending on the symptom scores. Parents with more anxiety and depression symptoms in pregnancy and postnatal alcohol problematic use have on average children with higher grades in English (ß = 0.01, SE = 0.004 and ß = 0.028, SE = 0.005 respectively). Anxiety and depression symptoms at age 8 and ADHD symptoms are not associated with English scores. And eating disorders symptoms were on the other side negatively associated (ß = −0.026, SE = 0.004 for symptoms during pregnancy, and ß = −0.013, SE = 0.005 for child’s age 8 symptoms), as expected and was found for maths and reading scores.

On average across symptoms and tests scores, an increase of one standard deviation in the parental symptom score is associated with a decrease of 0.02 SD of the child’s standardised test score, and on maximum 0.06 SD. For comparison, variables we include as covariates are more strongly associated. For example, being a boy is associated with an increase of 0.16SD in maths, a decrease of 0.12SD in reading, and an increase of 0.10SD in English. An increase of 1 SD in parental education is associated with an increase of 0.05 SD in maths and reading scores and about 0.03SD in English.

We ran the same regressions in the children-of-siblings subsamples, to investigate the specificity of this subset of the MoBa participants, and enable a comparison with a within-sibling model in the exact same subset. As expected given the smaller sample size, the estimates are less precise in the children-of-siblings subsamples, as reflected by wider confidence intervals (Fig. 2 – blue & Supplementary Tables 7 & 8). Overall, the associations are similar, but some point estimates are lower and many are no longer significantly different from zero. Amongst the previous 15 significant associations, only 5 are significant in the children-of-siblings subsample: anxiety and depression symptoms in pregnancy with maths scores, and eating disorders symptoms at both measurement ages with maths and reading scores.

### Within-children-of-sibling associations

After controlling for shared familial factors with a children-of-siblings design, no association between parental symptoms and children’s test scores was statistically significant (Fig. 2 – green and Supplementary Tables 7 & 8). For descriptive purposes, using the conventional *p* < 0.05 threshold *without* false discovery rate correction, few within-sibling associations reached “suggestive significance”. Amongst the 5 significant observational associations in the children-of-siblings subsample, 3 reached suggestive significance with a children-of-siblings design. Parental anxiety and depression symptoms in pregnancy were suggestively negatively associated with children’s maths scores (ß = −0.026, SE = 0.010). Eating disorder symptoms at age 8 were suggestively negatively associated with children’s maths and reading scores (ß = −0.078, SE = 0.027 and ß = −0.057, SE = 0.027 respectively). Additionally, eating disorder symptoms at age 8 were suggestively negatively associated with English test scores (ß = −0.075, SE = 0.027). For parental anxiety and depression symptoms and for eating disorders symptoms, controlling for shared familial factors by comparing children-of-siblings led to a reduction in the point estimates of the associations for symptoms during pregnancy. However, the point estimates with symptoms at age 8 were not reduced (the estimates are imprecise nevertheless).

We also ran all children-of-siblings analyses in same-sex sibships (note that for eating disorder symptoms, which were measured in mothers only, sibships are inherently same-sex). Estimates were similar to those presented here: none of the associations reached suggestive significance (Supplementary Tables 9–11 and Supplementary Fig. 4). Additionally, there was no visual evidence for non-linear relationships when plotting individual data and quartile trends.

## Discussion

Parents who reported more symptoms of anxiety, depression, ADHD, or eating disorders tended to have children scoring lower on standardised tests of mathematics and reading in 5th grade (ten-year-olds). After controlling for shared familial factors by comparing up to 18,000 children of siblings, no significant associations remain, providing no support for a causal impact of parental mental health on children’s academic achievement. However, ascertainment differences between the overall MoBa sample and the children-of-siblings samples hinder strong generalisations about the lack of causal mechanisms, as some associations might remain statistically significant in a larger, more representative children-of-siblings sample. Nonetheless, our study suggests an increase of one SD in parental mental health scores would decrease their children’s test score by 0.08 SD *at most*, in the case of eating disorder symptoms.

Importantly, parental symptoms experienced close in time to the child’s academic test may matter more than parental symptoms during pregnancy. Children born to mothers with an eating disorder during pregnancy had on average lower test scores across domains (β from − 0.04 to −0.02), but these associations completely disappeared (β ~ 0) after controlling for shared familial factors, suggesting that they are explained by factors shared within the extended family. In contrast, this attenuation was not observed for maternal eating disorder symptoms measured when children were 8: After controlling for shared familial factors, parental symptoms remain suggestively associated with lower maths and English test scores two years later (βs ~ 0.08). A similar pattern, though less pronounced, is also visible for anxiety and depression symptoms. One possible interpretation is that parental symptoms closer in time to testing may have a stronger influence on children’s academic functioning, for example, through more immediate disruptions in the home environment or reduced parental availability. Alternatively, this pattern may reflect the presence of additional unaccounted confounding when parental symptoms are measured later in life. As siblings’ environments diverge over time, fewer factors may be shared within sibling pairs, and fewer factors might therefore be adjusted for in the children-of-siblings design. It is also possible that child characteristics influence both parental mental health and academic outcomes, contributing to these associations. This timing effect warrants further investigation using other cohorts and designs.

We find that the association of children’s test scores with parental mental health depends on the type of symptoms. As we do not find strong evidence for a causal effect for any of these associations, it is likely that differences between symptoms are due to different symptoms relating differently to shared familial factors, such as socioeconomic status or shared genetics. For example, parents who score higher than average on ADHD symptoms tend to have children who also score higher on ADHD symptoms. Previous research has shown that this is mostly due to the transmission of genetic liability, rather than the effect of being exposed to parental ADHD [12, 37, 51]. As childhood ADHD generally decreases academic performance [46], children of parents with ADHD are likely at risk for poor educational outcomes not because of parental exposure, but because of their own inherited traits genetically associated with ADHD. Alternatively, anxiety is thought to be transmitted to their children mostly by socialisation [2, 20], children of anxious parents are more likely to have lower scores due to environmental factors shared with their parents, than to shared genetics. Additionally, we cannot rule out that differences in measurement of parental mental health (some disorders could be better captured than others) might affect our comparison of parental disorders.

Some surprising positive observational associations emerged for English test scores: Higher parental symptoms of anxiety and depression in pregnancy, as well as higher symptoms of problematic alcohol use, were associated with slightly higher scores in English. These associations may reflect confounding rather than causal effects. One possible explanation is that lower socioeconomic status is associated both with higher parental symptoms and with greater children’s screen use, which may increase exposure to English-language content [11, 33]. For problematic alcohol use, an additional explanation is its non-linear association with socioeconomic status: both alcohol consumption and active exposure to English may be more common in higher-educated families [8, 24, 29]. In any case, both associations disappear in the within-family analysis, suggesting that neither reflects a causal effect of parental symptoms on children’s English scores.

Our study examines the effect of parental self-reported symptoms in a population-based sample of Norwegian parents, capturing variation across the full distribution of mental health symptoms. Compared to diagnostic-based designs, these measures are less directly tied to factors influencing who receives a diagnosis (e.g., severity thresholds or healthcare access). However, more severe cases that would be captured in registry-based diagnoses may be underrepresented in our sample (see discussion below). Our conclusions are nevertheless consistent with a recent study in the full (Norwegian) population using diagnosed internalising disorders [47]. Nordmo et al., compare test scores of 16-year-old siblings differentially exposed to their parents’ internalising disorders. They found that only exposure to parental psychopathology close to academic testing appears to have an effect, of small size. The impact of exposure to other diagnosed parental psychopathology on academic achievement should be further investigated using genetically-informed designs [44].

There are limitations to our study and its interpretation. First, despite a relatively big sample size, our sample is not fully representative of the Norwegian population. This is evident from the mean standardised test scores in MoBa, which are 0.10–0.26 SD above the population mean, and those in the children-of-siblings subsample, which are 0.10–0.35 SD above the population mean. Moreover, we see in Fig. 2 that the full MoBa and children-of-siblings subsample sometimes give different point estimates of the observational associations; see for example parental ADHD symptoms (but note the confidence intervals overlap). Ascertainment bias might be a concern, as parents with mental disorders or with lower SES are less likely to participate in surveys [36]. This selection may also limit the representation of more severe psychopathology in our sample. Exposure to more severe parental psychopathology might have stronger effects on children’s test scores, as it may involve additional adversities for children, such as parental divorce or absence due to hospitalization [3, 23, 54]. We therefore cannot rule out the possibility that associations differ at more extreme levels of parental psychopathology, which were not specifically examined here and may be less precisely captured in questionnaire-based measures. We recommend replication in an even larger, more representative sample. Reweighting data to correct for ascertainment bias might also be an option [7, 59], once such weights become available for MoBa [55].

Second, our children-of-siblings design is also subject to the same limitations as typical sibling comparison designs [61]. Random measurement errors of the parental symptoms might accentuate the attenuation of the within-sibling effect [25, 26]. How within-sibling designs are affected by ascertainment bias depends on the factors leading to selection. If those are stable and shared across siblings, they will be adjusted for by design [41], but if those are not shared within siblings, a within-sibling design might amplify the bias due to these factors [61]. Confounders not shared between siblings, such as individual experiences and non-shared genetics, might still confound association estimates [56].

Third, our findings are based on a Norwegian cohort and should be interpreted in this context. Differences in a wide range of social support systems, including preschool and education, access to (mental) health care, benefits for seriously ill parents, and general social support for families, may influence the extent to which parental mental health relates to children’s academic achievement. Therefore, generalisability to other contexts may be limited. Finally, we recommend investigating whether exposure to parental psychopathology impacts offspring’s educational outcomes later on, up to their highest obtained degree. Our observed parental effects at age 10 that seem negligible may accumulate, or parental effects may be larger in secondary and tertiary education [12, 14].

## Conclusion

While our study has limitations, like healthy volunteer bias and imperfect control for confounding, that prevent us from firmly establishing a causal relationship, our findings suggest that parents’ mental health symptoms are unlikely to have a significant average impact on their children’s academic performance. We estimate that such effects, if they exist, are likely to be small, explaining little of the variation among children in their standardised test scores. While our results do not diminish the importance of addressing mental health concerns in parents, they do suggest that improving parental mental health in general may not in itself lead to improvements in children’s educational outcomes.

## Supplementary Information

Below is the link to the electronic supplementary material.

Supplementary Material 1 (XLSX 320 KB) 

Supplementary Material 2 (DOCX 940 KB)

## Data Availability

Data availability for research purpose is subject to strict Norwegian privacy regulations, see https://www.fhi.no/en/studies/moba/for-forskere-artikler/research-and-data-access/and https://www.ssb.no/en/data-til-forskning/utlan-av-data-til-forskere.
