# Supplementary material for: No evidence of parental mental health influencing children’s academic achievement: a within-family study
Source: Eur Child Adolesc Psychiatry. 2026 Jun 24;35(11):3427–38. doi: 10.1007/s00787-026-03100-9 (PMC13710637; doi:10.1007/s00787-026-03100-9)
Supplement: Supplementary file 1 [file 787_2026_3100_MOESM2_ESM.docx]

Supplementary Note and Figures

[Deviation from the preregistration	1](#_Toc231225806)

[The role of parental genetic liability	1](#_Toc231225807)

[Parental mental health measures	2](#_Toc231225808)

[Adjusting for parental education	4](#_Toc231225809)

[Supplementary Figure 1. Causal diagram illustrating shared and nonshared confounders in the children-of-sibling design.	5](#_Toc231225810)

[Supplementary Figure 2. Flowchart of study sample	6](#_Toc231225811)

[Supplementary Figure 3. Test information functions and test characteristics curves for the nominal response models.	7](#_Toc231225812)

[Supplementary Figure 4. Same-sex siblings models.	8](#_Toc231225813)

[Supplementary references	9](#_Toc231225814)

# Deviation from the preregistration

Unlike preregistered we did not include a random effect for the individual in regressions (1) and (2), as we do not have repeated measured and one individual is only included within one family. Additional to preregistered analyses, we also run analyses including siblings from opposite sex. In these analyses, we include the sex of the parent as a covariate.

# The role of parental genetic liability

Parental genetic liability could influence child outcomes in several ways:

- Via parental symptoms, in which case genetic liability precedes the causal chain so does not bias our estimate of symptom effects.
- Via the child’s genetic liability, where parental genes influence both their own symptoms and their child’s genetic liability, which affect childhood academic achievement, producing a spurious association between symptoms and achievement.
- Via other parental traits, for example genetic liability influencing residential choices or socioeconomic outcomes, which in turn affect both parental symptoms and child outcomes. (See below section about “adjusting for parental education” aimed at disentangling such a possibility).

Note that the last two cases are examples of gene–environment correlation, where parental genetic liability acts as a confounder of the association between parental symptoms and children’s academic achievement.

The children-of-sibling design adjusts for confounding by the genetic liability shared by parental siblings (genetic liability at the level of the grandparents, part of U_a_ in Supplementary Figure 1.). It does not adjust for the genetic liability that is not shared between siblings, but that is nonetheless shared between parents and children (part of U_b_ in Supplementary Figure 1.). Hence, a children-of -sibling design cannot fully adjust for genetic confounding and is not able to differentiate between the above case scenarii.

# Parental mental health measures

##### Anxiety and depression

To measure parents’ anxiety and depression symptoms we use the Hopkins Symptoms Checklist (SCL)^1^, which assesses self-reported symptoms during the last 2 weeks. We looked at the first assessment (15th week of pregnancy) and at the closest measure to the national tests (questionnaire sent when the child is 8 years). For fathers, SCL-90 was only measured in the first questionnaire (15th week of pregnancy) and in 2015. For mothers, a short version of the original SCL-90 was assessed in most questionnaires^2^: a subset of 5 items was asked to mothers during pregnancy, and 8 items at age 8 (4 items capture symptoms of anxiety and 4 depression). We use SCL-5 at 15 weeks of pregnancy and SCL-8 at age 8 and 2015, for both fathers and mothers.

##### ADHD

We use the Adult ADHD Self-Report Scale (ASRS Screener), which consists of 6 questions^3,4^. It was reported for mothers in the questionnaire Q6 (36 months-old) and in the latest version of the father questionnaire at 15-weeks pregnancy.

##### Problematic alcohol consumption

Alcohol use was measured in MoBA using the Alcohol Use Disorders Identification Test (AUDIT), reported for mothers in the questionnaire at age 8, and for fathers in the 2015 questionnaire for fathers. We use the 7 items of the screening tool which relate to problematic use (AUDIT-P)^5,6^.

##### Eating Disorders

Eating disorders were only assessed in mothers^7^. We measure eating disorders symptoms using 14 items on eating behaviours reported at 15-weeks of pregnancy and 12 items in the age 8 questionnaire. We computed a continuous eating disorder risk score using item response theory on the DSM-IV eating disorder symptoms. In Stata 17, we computed a nominal response model for each time point, we report parameters estimates in **Supplementary Tables 10 & 11** and test characteristics curve and test information functions in **Supplementary Figure 2**.

# Adjusting for parental education

We investigated the attenuation of the within-sibling effect when taking into account parental education. Educational attainment of the parents is an important potential confounding or mediating factor that might not be adjusted for in the within-sibling design (not shared between the siblings).

Parental educational attainment was obtained via linkage to the National Educational database. Assuming parental education is mostly stable over time, we used the measure of educational attainment taken in 2023, most recent year available. Parental educational attainment is coded using the International Standard Classification of Education (ISCED) standard, which we converted into the expected number of years needed to achieve this level of attainment (following^8^).

To assess the role of mental health above the role of educational attainment, we estimated model 2 adding both parents’ educational attainment as an additional covariate. Unsurprisingly, none of the within-sibling associations between parental symptoms and children’s test scores are significant when additionally adjusting for parental education (Supplementary Tables 2 & 3). Eating disorders symptoms at age 8 is nevertheless still suggestively associated with maths test scores (ß = -0.058, SE = 0.028).

# Supplementary Figure 1. Causal diagram illustrating shared and nonshared confounders in the children-of-sibling design.

In the context of our study, the exposures X_1_ and X_2_ represent the parental symptoms for sibling 1 and sibling 2, and the outcomes Y_1_ and Y_2_ are the test scores of the siblings’ children. Two types of confounding factors might affect the association between X and Y: U_a_ and U_b_. U_a_ are shared familial confounding factors: factors shared between the siblings in the parental generation, which includes **roughly 50% of their segregating genes and aspects of their childhood family environment.** U_b_ are confounding factors that are not shared between the siblings in the parental generations: the remaining 50% of their genes, their adult environment, etc. These are factors are unique to the sibling and might affect both the sibling’s symptoms and their children’s test scores. The children-of-sibling design allows to get an estimate for the association between X and Y while adjusting for U_a_, but it does not adjust for U_b_.

# Supplementary Figure 2. Flowchart of study sample

# Supplementary Figure 3. Test information functions and test characteristics curves for the nominal response models.

A. **Eating disorders symptoms during pregnancy.**

B. **Eating disorder symptoms at child’s age 8**

# Supplementary Figure 4. Same-sex siblings models.

Coefficients as estimated with a linear regression in the children-of-siblings subsamples (blue), and with the children-of-sibling model (green). Bars are 95% CIs.

A. **Only fathers.**

B. **Only mothers.**

# Supplementary references

1.	Derogatis, L. R., Lipman, R. S. & Covi, L. SCL-90: an outpatient psychiatric rating scale--preliminary report. *Psychopharmacol. Bull.* **9**, 13–28 (1973).

2.	Tambs, K. & Røysamb, E. Selection of questions to short-form versions of original psychometric instruments in MoBa. *Nor. Epidemiol.* **24**, (2014).

3.	Kessler, R. C. *et al.* The World Health Organization Adult ADHD Self-Report Scale (ASRS): a short screening scale for use in the general population. *Psychol. Med.* **35**, 245–256 (2005).

4.	Kessler, R. C. *et al.* Validity of the World Health Organization Adult ADHD Self-Report Scale (ASRS) Screener in a representative sample of health plan members. *Int. J. Methods Psychiatr. Res.* **16**, 52–65 (2007).

5.	DeRoo, L. A. Maternal alcohol consumption in the Norwegian Mother and Child Cohort Study (MoBa) – Research opportunities. *Nor. Epidemiol.* **24**, (2014).

6.	Saunders, J. B., Aasland, O. G., Babor, T. F., de la Fuente, J. R. & Grant, M. Development of the Alcohol Use Disorders Identification Test (AUDIT): WHO Collaborative Project on Early Detection of Persons with Harmful Alcohol Consumption--II. *Addict. Abingdon Engl.* **88**, 791–804 (1993).

7.	Bulik, C. M. *et al.* Patterns of remission, continuation and incidence of broadly defined eating disorders during early pregnancy in the Norwegian Mother and Child Cohort Study (MoBa). *Psychol. Med.* **37**, 1109–1118 (2007).

8.	Isungset, M. A. *et al.* Social and Genetic Effects on Educational Performance in Early Adolescence. Working Paper at https://doi.org/10.3386/w28498 (2021).

9.	Petersen, A. H. & Lange, T. What Is the Causal Interpretation of Sibling Comparison Designs? *Epidemiology* **31**, 75–81 (2020).
